# Tracking viral control in adolescents on antiretroviral therapy in Lusaka, Zambia: A retrospective cohort analysis

**DOI:** 10.4102/sajhivmed.v26i1.1665

**Published:** 2025-04-24

**Authors:** Kaala Moomba, Talitha Crowley, Brian van Wyk

**Affiliations:** 1 School of Public Health, Faculty of Community and Health Sciences, University of the Western Cape, Cape Town, South Africa; 2 School of Nursing, Faculty of Community and Health Sciences, University of the Western Cape, Cape Town, South Africa

**Keywords:** adolescents, antiretroviral therapy, cohort, viral suppression, retrospective, Zambia

## Abstract

**Background:**

In 2023, an estimated 39.9 million people globally were living with HIV, of which 1.55 million were adolescents aged 10–19 years. The 2021 Zambia HIV Impact Assessment revealed lower viral suppression rates in adolescents (15–24 years old) compared to adults on antiretroviral therapy (ART). Lusaka District, Zambia, has the highest number of adolescents on ART, with a 15.1% HIV prevalence in 2018.

**Objectives:**

To determine the prevalence and factors associated with viral suppression among adolescents living with HIV (10–19 years) on ART in Lusaka District, Zambia.

**Method:**

A retrospective cohort analysis was done of 3409 adolescents on ART at public health facilities in Lusaka from January 2023 to December 2023, and who had viral loads recorded. Socio-demographic, clinical, treatment and behavioural data were extracted from electronic health records and analysed using SPSS version 29.

**Results:**

The adolescent cohort in Lusaka achieved 91.8% viral suppression rate (< 1000 copies/mL), with 79% fully suppressed (< 50 copies/mL). In multivariate analysis, older adolescents (15–19 years) had lower odds of suppression compared to younger adolescents (10–14 years) (adjusted odds ratio [AOR] = 1.79; confidence interval [CI] : 1.32–2.43). Higher odds of viral suppression were linked to first-line dolutegravir regimen (AOR = 5.12; CI: 3.23–8.11) and optimal adherence (AOR = 1.89; CI: 1.03–3.47), while regimen switches reduced the odds of viral suppression (AOR = 0.60; CI: 0.45–0.80).

**Conclusion:**

Zambia reached the previous UNAIDS 90-90-90 targets with a viral suppression rate of 91.8%. However, to reach the revised 95% target by 2030, tailored interventions should be implemented to improve adherence and retention in care, particularly for older adolescents.

**What this study adds:** This study adds insights into adolescent viral suppression rates, highlighting disparities by age and treatment regimen. The study provides evidence that first-line dolutegravir regimens and optimal adherence significantly enhance viral suppression.

## Introduction

The HIV pandemic remains a significant global health challenge, particularly affecting adolescents aged 10–19 years. In 2023 an estimated 39.9 million (confidence limits: 36.1–44.6 million) people were living with HIV, of which approximately 1.55 million (1.2–1.9 million) were adolescents.^1^ Adolescents and young adults aged 10 years to 24 years continue to be disproportionately affected by HIV with 360 000 (240 000–480 000) new HIV infections, of which 140 000 (39 000–240 000) were older adolescents aged 15 years and 19 years.^1^ This population faces a significantly higher risk of antiretroviral therapy (ART) non-adherence, increasing their susceptibility to mortality and morbidity compared to other age groups.^2^

The Joint United Nations Agency on HIV/AIDS (UNAIDS) 95-95-95 global target, which states that 95% of people living with HIV (PLWH) should know their status, 95% of those who know their status should receive ART, and 95% of those in care should have a suppressed viral load (VL) by 2030 is recommended for low- and middle-income countries as part of a fast-track strategy to end the HIV epidemic.^3^ Antiretroviral therapy is crucial for improving the duration and quality of life of PLWH by suppressing the virus, reducing morbidity and mortality and enhancing overall health.^4^

The World Health Organization (WHO) recommends that all PLWH on ART undergo routine VL testing to achieve these global targets. At local level, VL monitoring is critical for measuring ART efficacy, early detection of drug resistance, improving clinical outcomes, improving treatment adherence and reducing HIV transmission.^5^ Zambia has adopted the WHO recommendation of regular monitoring of VL, with the first VL test at 6 months and the second 12 months after starting ART, and annual testing thereafter.^5^

The WHO recommends three key categories for HIV VL assessments: unsuppressed (> 1000 copies/mL); suppressed (< 1000 copies/mL); and fully suppressed or undetectable (VL not detected by test or sample type used) < 50 copies/mL.^6^ Zambia adopted the WHO guidelines that set viral suppression as not suppressed at ≥ 1000 copies/mL and suppressed as < 1000 copies/mL.^7,8^ However, while Zambia has set viral suppression at < 1000 copies/mL, it is important to note that maintaining VL levels below 200 copies/mL is critical to effectively preventing HIV transmission, aligning with the ‘Undetectable = Untransmittable (U = U)’ principle. This is particularly relevant among adolescents, where ensuring sustained viral suppression at this lower threshold can significantly reduce transmission risks and improve long-term health outcomes.^9^

Adolescents living with HIV (ALHIV) face unique challenges that are associated with rapid physical, psychological and physiological changes during adolescence that impact behaviour negatively on treatment adherence,^10,11^ which in turn leads to mental health problems,^12,13^ reduced engagement with healthcare and lower rates of viral suppression. However, the adolescent population is often overlooked because programmatic reporting does not use the WHO definition of adolescents (10–19 years old) and instead classifies and reports them under paediatrics (0–14 years old) or adult (15 years old and older) populations.

Viral suppression in PLWH is influenced by a complex interplay of socio-demographic, clinical, treatment and behavioural factors. Existing literature emphasises the role of socio-demographic characteristics, such as (current) age, age at ART initiation and gender in determining viral suppression outcomes.^14,15^ Several studies report lower rates of viral suppression in older adolescents compared to younger adolescents due to treatment fatigue (being on ART from childhood), and not being adequately prepared to transition to adult care and self-management.^16,17^ Similar conflicting results are reported for female versus male adolescents due to gender differences in health-seeking behaviour,^18^ as well as higher infection rates amongst adolescent girls compared to boys.^1^

Clinical factors such as duration on ART, and baseline CD4 count and WHO clinical stage have been found to be strong predictors of viral suppression.^14,17,19,20^ Studies have shown that adolescents who start ART with a less advanced immunodeficiency (CD4 > 200 cells/mm^3^ – 350 cells/mm^3^) appear to have better virological outcomes than adolescents who start with a more severe immunodeficiency (CD4 count < 200 cells/mm^3^).^19,21,22,23,24^ However, other studies found that higher CD4 counts were not associated with viral suppression.^25,26,27^ Various studies have reported variations around the duration on ART with viral suppression, where longer duration on ART is expected to be associated with viral suppression.^28^

Treatment characteristics such as the choice of antiretroviral regimen and changes to a more efficacious regimen are vital for achieving viral suppression.^29,30,31^ Following the WHO recommendion for use of dolutegravir (DTG) as the preferred core agent in ART for first and second-line regimen,^32^ studies have reported improved viral suppression in PLWH which includes children and adolescents.^33^

Behavioural characteristics, such as optimal adherence to medication and retention in care, are strongly correlated with viral suppression.^34^ Optimal adherence is defined as taking at least 95% medications at prescribed times and frequencies (at least 95%),^35,36^ while retention in care is a patient’s regular engagement with medical care at a health care facility after initial entry into HIV clinical care as set out with appointments schedules.^37^ According to Zambian guidelines, one is deemed out of care (not retained) once they miss a pharmacy refill for 30 days or more.^7^

Whereas the risk factors influencing viral suppression among ALHIV are known and clearly illustrated in the literature, these factors may differ in various contexts. It is therefore essential to identify the specific set of factors associated with viral suppression that are prevalent in ALHIV in Zambia, as this information is crucial for tailoring interventions and programmes to improve adherence and viral suppression as progressive steps to reach the UNAIDS 95-95-95 targets by 2030.

### Objectives

The objective of the current study was to determine the prevalence and factors associated with viral suppression among ALHIV (10–19 years) on ART from January 2023 to December 2023 in Lusaka District, Zambia.

## Research methods and design

### Study design

We conducted a retrospective study of routinely collected data from ALHIV on ART from January 2023 to December 2023 to determine the prevalence and factors associated with viral suppression in the Lusaka District. Routine socio-demographic, clinical, treatment, and behavioural data were collected for adolescents aged 10 years to 19 years who were enrolled in public health facilities providing ART in Lusaka District. Behavioural data included measures of optimal adherence (assessed through pharmacy refill data) and retention in care (assessed through missed appointment tracking).

### Study context

Lusaka District is located in the southern part of Zambia’s Central Province and serves as the capital city of the country. Health services in the district are provided at different levels of care. The levels of care in the public sector range from health posts (lowest level) to specialised hospitals which are designated third-level hospitals (highest level). The master facility list shows that Lusaka District has 36 health posts, 31 health centres, five level one hospitals and eight level three (specialised) hospitals. Health posts and health centres mainly provide primary healthcare services which include HIV services. First level hospitals act as referral facilities for health posts and health centres and also provide HIV services. Third level hospitals are specialised facilities which mainly handle complicated cases referred to from lower levels of care.

### Study population and sampling

The study population consisted of all adolescents aged 10–19 years receiving ART and accessing ART services in Lusaka District in 2023.

### Data collection

Routine data were extracted from an electronic health record (EHR) called SmartCare. SmartCare is a commonly used EHR in public health facilities across Zambia. Its implementation varies by facility: some operate on an electronic last or e-last model, where data entry is completed retrospectively by designated data entry personnel, while others use an ‘electronic first’ (e-first) approach, where healthcare providers at all service points within the facility input patient data in real time.

Study data were extracted in August 2024 and consisted of anonymised data identified by unique patient identifiers. Data included demographic, clinical, treatment, and behavioural data collected during routine clinical visits for patient management and monitoring of all patients receiving ART. All ART data are captured using SmartCare and therefore any missing data were unable to be retrieved. Viral load was the primary outcome variable: VL < 50 RNA copies/mL was categorised as ‘fully suppressed’; VL > 50 copies/mL – 999 copies/mL was categorised as suppressed; and VL > 1000 copies/mL was categorised as ‘unsuppressed’ according to national clinical ART guidelines.^7^ In this study, ‘viral suppression’ applies to all participants who had a VL of < 1000 copies/mL most recently recorded in SmartCare.

Socio-demographic variables included gender, current age, and age at ART initiation. Clinical characteristics included baseline WHO clinical stage, CD4 count (baseline and current), pregnancy, lactation, and history of active tuberculosis (TB). Treatment characteristics included current ART regimen, current ARV regimen class, change in ART regimen, and use of TB preventive therapy. Behavioural characteristics included retention in care and optimal adherence to ART. In the current study, retention in care was defined as currently being active on ART, and optimal adherence to ART is defined as missing a medication refill appointment by no more than 2 days.

### Data analysis

The extracted data were entered into an Excel spreadsheet, cleaned, and then imported into the IBM Statistical Package for the Social Sciences (SPSS) for Mac, Version 29.0.2.0 (IBM Corp., Armonk, New York). For inferential analysis, variables with missing values were treated using complete-case analysis, with cases with missing data excluded from the analysis. For categorical variables, descriptive frequency tables were created to describe the socio-demographic, clinical, behavioural, and treatment characteristics of the study population. Bivariate analysis was used to determine the significance and strength of associations between the independent variables and the outcome variable (VL < 1000 copies/mL). Chi-square tests and crude odds ratios were used to examine and quantify associations, with significance set at *P* < 0.05. Variables that showed significant associations with viral suppression during bivariate analysis, at a significance level of 5%, were included in the multivariate analysis to identify factors independently associated with viral suppression, and presented as adjusted odds ratio (AOR) with 95% confidence intervals (95% CI).

### Ethical considerations

Ethical clearance was obtained from the University of the Western Cape Biomedical Research Ethics Committee (reference number: BM24/3/4), and the Mulungushi University School of Medicine Ethics Committee (reference number: SMHS-MU2-2024-04) and the Zambia National Health Research Authority (reference number: NHRA1186/15/05/2024). Approval for the study and access to the data were obtained from the Zambia Ministry of Health (reference number: MH/101/22/3). During data extraction, the adolescents’ unique identities, such as ART number, identity number, first and last name, were excluded to ensure complete anonymity and protection of personal information.

## Results

### Realisation of sample

**FIGURE 1:**
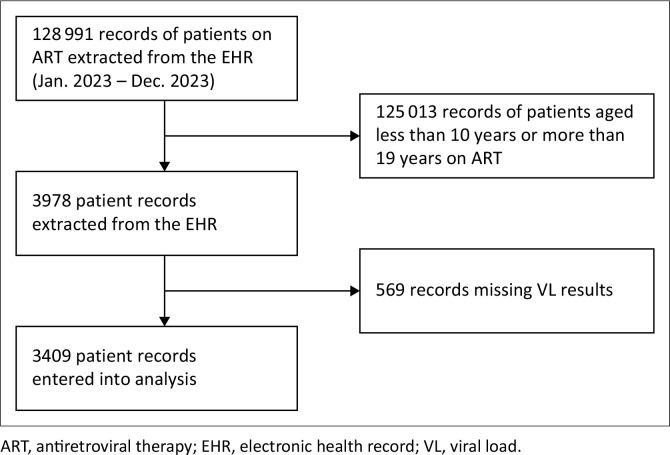
Flow chart of participants selected in the study.

**FIGURE 2:**
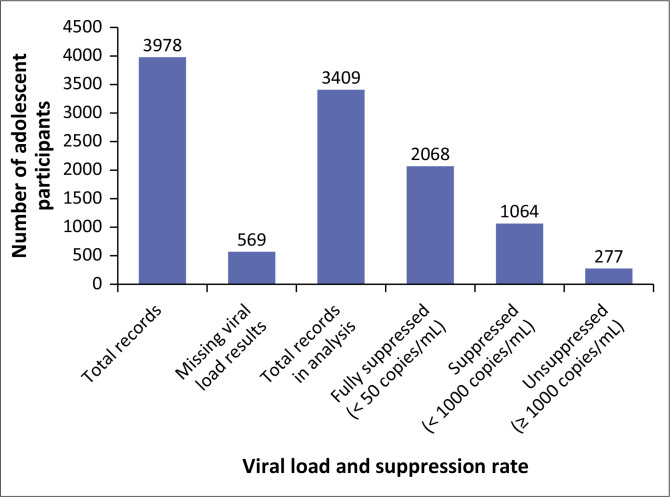
Distribution of viral load records and suppression status.

Records of 128 991 PLWH attending ART services in health facilities in Lusaka District from January 2023 to December 2023 were obtained from the EHR. Figure 1 shows that of 128 991 records, 125 013 were records of PLWH aged less than 10 years and more than 19 years who were in care in 2023, while 3978 records were of adolescents aged 10 years to 19 years during 2023. 569 records of the 3978 adolescents had missing VL results in the EHR (see Figure 2).

### Socio-demographic, clinical, treatment and behavioural characteristics

Table 1 shows the socio-demographic, clinical, treatment and behavioural characteristics of ALHIV enrolled in the ART programme in public health facilities in Lusaka District, Zambia.

**TABLE 1:** Determinants of viral suppression (< 1000 copies/mL) among adolescents on antiretroviral therapy in the Lusaka District, 2023 (*N* = 3409).

Characteristics	Total		Viral suppression				*P*
			Yes		No		
	*n*	%	*n*	%	*n*	%	
**Total**	3409	-	3132	91.8	277	9.2	-
**Age (years)**	-	-	-	-	-	-	< 0.010 *
10–14	1384	40.6	1303	94.1	81	5.9	-
15–19	2025	59.4	1829	90.3	196	9.7	-
**Gender**	-	-	-	-	-	-	**0.539**
Male	1589	46.6	1455	91.6	134	8.4	-
Female	1820	53.4	1677	92.1	143	7.9	-
**Age at ART initiation (years)**	-	-	-	-	-	-	**0.200**
0–4	1061	31.1	984	92.4	77	7.3	-
5–9	1178	34.6	1084	92.0	94	8	-
10–14	933	27.3	854	91.5	79	8.5	-
15–19	237	7.0	210	88.6	27	11.4	-
**Duration on ART (months)**	-	-	-	-	-	-	**0.534**
6–12	8	0.2	7	87.5	1	12.5	-
13–24	112	3.3	100	89.3	12	10.7	-
> 24	3289	96.5	3025	92.0	264	8.0	-
**Current ART regimen**	-	-	-	-	-	-	< 0.010*
First-line	3241	95.1	3027	93.4	214	6.6	-
Second-line	146	4.3	91	62.3	55	37.7	-
Unclassified (Incomplete regimen)	22	0.6	14	63.6	8	36.4	-
**Current ARV regimen class**	-	-	-	-	-	-	< 0.010*
NNRTI	6	0.2	4	66.7	2	33.3	-
DTG-based	3351	98.3	3102	92.6	249	7.4	-
PI-based	30	1.0	12	40.0	18	60.0	-
Unclassified (Incomplete regimen)	22	0.5	14	63.6	8	36.4	-
**Change in ART regimen**	-	-	-	-	-	-	< 0.010*
Yes	1030	30.2	912	88.5	118	11.5	-
No	2379	69.8	2220	93.3	159	6.7	-
**Pregnant† (*N* = 1820)**	-	-	-	-	-	-	0.010*
Yes	17	1.0	13	76.5	4	23.5	-
No	30	1.6	25	83.3	5	16.7	-
Unknown	1773	97.4	1639	92.4	134	7.6	-
**Breastfeeding (*N* = 1820)**	-	-	-	-	-	-	**0.559**
Yes	4	0.2	4	100.0	0	0.0	-
No	1816	99.8	1673	92.1	143	7.9	-
**Baseline CD4 count (cells/mm^3^)† (*N* = 1671)**	-	-	-	-	-	-	0.011*
< 200	150	9.0	129	86.0	21	14.0	-
200–349	217	13.0	197	90.8	20	9.2	-
≥ 350	1304	78.0	1211	92.9	93	7.1	-
**Latest CD4 count (cells/mm^3^)† (*N* = 2139)**	-	-	-	-	-	-	< 0.010*
< 200	74	3.4	60	81.1	14	18.9	-
200–349	168	7.9	147	87.5	21	12.5	-
≥ 350	1897	88.7	1758	92.7	139	7.3	-
**Current WHO stage (*N* = 2971)**	-	-	-	-	-	-	**0.486**
Stage I	2935	98.7	2701	92.0	234	8.0	-
Stage II	21	1.0	18	85.7	3	14.3	-
Stage III	14	0.3	14	100.0	0	0.0	-
Stage IV	1	0.0	1	100.0	0	0.0	-
**Received TB prophylaxis**	-	-	-	-	-	-	**0.053**
Yes	2747	80.6	2536	92.3	211	7.7	-
No	662	19.4	596	90.0	66	10.0	-
**History of active TB**	-	-	-	-	-	-	**0.536**
Yes	159	4.7	144	90.6	15	9.4	-
No	3250	95.3	2988	91.9	262	8.1	-
**Retention in care**	-	-	-	-	-	-	0.019*
Yes (In care)	3132	91.9	1112	93.4	2020	91.1	-
No (IIT, TO, died)	277	9.1	79	6.6	198	8.9	-
**Optimal adherence (*N* = 2940)**	-	-	-	-	-	-	0.044*
Yes	2837	96.5	2609	92.0	228	8.0	-
No	103	3.5	89	86.4	14	13.6	-

ART, antiretroviral therapy; ARV, antiretroviral drugs; CD4, cluster of differentiation 4; DTG, dolutegravir; IIT, Interruption in treatment; NNRTI, non-nucleoside reverse transcriptase inhibitor; PI, protease inhibitor; TB, tuberculosis; TO, transfer out.

† , Variables not included in the regression model due to very small sample sizes.

* , Statistically significant at *p* < 0.05

This study involved 3409 adolescents (aged 10 years to 19 years) with a mean age of 14.97 years (standard deviation [s.d.] = 1.15); of these, 53% (1820) were female. The mean age at ART initiation was 7.51 years (s.d. = 1.15). The majority of participants (2935, 98.7%) were at WHO clinical stage 1. Most participants (1897, 78% and 1897, 88.6%) had both baseline and recent CD4 counts ≥ 350 cells/mm^3^. The vast majority of participants (3241, 95.1%) received first-line therapy, and were on a DTG-based regimen (3351, 98%). Majority of participants (3132, 91.9%) remained in care, while 9.1% (277) experienced an interruption in treatment (IIT), transferred out or died during the study period. Most participants (2837, 96.5%) demonstrated optimal adherence to their ART, as defined by not missing a pharmacy refill by more than 2 days.

### Determinants of viral suppression

The overall viral suppression was 91.8% (< 1000 copies/mL) while fully suppressed was 66% (< 50 copies/mL). Table 2 shows that in bivariate analysis, the odds of viral suppression were significantly higher (94% vs 90%; *P* < 0.01) in individuals aged 10 years to 14 years than those aged 15 to 19 years (crude OR = 1.79, 95% CI = 1.32 – 2.42). After adjusting for other variables, the significant association between viral suppression and age remained (adjusted OR [AOR] = 1.79, 95% CI = 1.32 – 2.43) (see Table 2). No statistically significant associations were found between viral suppression and gender (*P* = 0.539), and age at initiation of ART (*P* = 0.2) (see Table 1).

**TABLE 2:** Determinants of viral suppression of among adolescents (10–19 years) on antiretroviral therapy in Lusaka District, 2023.

Characteristics	Total	Crude OR	95% CI	Adjusted OR	95% CI
**Age (years)**					
10–14	1384	Ref	-	Ref	-
15–19	2025	1.74	1.32–2.26*	1.69	1.25–2.29*
**Current ART regimen**					
First-line	3241	Ref	-	Ref	-
Second-line	146	8.55	5.9–12.28*	6.14	3.86–9.48*
**Current ARV regimen class**					
NNRTI	6	Ref	-	Ref	-
DTG-based	3351	0.16	0.29–0.88*	0.39	0.56–2.74
PI-based	30	-	-	1.27	0.16–10.10
**Optimal adherence**					
Yes	2837	Ref	-	Ref	-
No	103	**1.80**	1.01–3.21*	1.82	0.97–3.41
**Retention in care**					
Yes	3132	Ref	-	Ref	-
No	277	1.38	1.05–1.81	1.25	0.93–1.69

ARV, antiretroviral drugs; CI, confidence interval; OR, odds ratio; DTG, dolutegravir; NNRTI, non-nucleoside reverse transcriptase inhibitor; PI, protease inhibitor.

* , Statistically significant at *p* < 0.05

There were statistically significant associations between viral suppression and pregnancy (*P* = 0.010), baseline CD4 count (*P* = 0.011) and latest CD4 count (*P* < 0.01). However, CD4 counts were only recorded for 42% and 54% of participants at baseline and current (observation year), respectively, while pregnancy and breastfeeding were documented for only 3% and 53% of all female participants and were therefore excluded from the multivariate analysis. No statistically significant associations were identified between viral suppression and clinical factors such as history of active TB (*P* = 0.145), current WHO stage (*P* = 0.486), and breastfeeding (*P* = 0.559) (see Table 1).

Statistically significant associations between viral suppression and treatment factors, including current ART regimen (*P* < 0.01), current ARV regimen class (*P* < 0.01) and change in ART regimen (*P* < 0.01) were observed. In the multivariate logistic regression, the relationship between viral suppression and current ART regimen was statistically significant (AOR = 6.14, 95% CI = 3.86 – 9.48), while the relationship between viral suppression and current ART regimen class did not retain statistical significance. In multivariate logistic regression, the relationship between viral suppression and change in ART regimen retained statistical significance (AOR = 0.62, 95% CI = 0.46 – 0.83).

The relationship between viral suppression and behavioural characteristics optimal adherence (AOR = 1.82, 95% CI = 0.97 – 3.41) and retention in care (AOR = 1.25, 95% CI = 0.93 – 1.69) did not retain statistical significance after adjusting for potential confounders.

## Discussion

Lusaka District made significant progress towards achieving the previous goal of 90-90-90 set by UNAIDS, reaching a 91.8% viral suppression rate for ALHIV, based on a threshold of < 1000 copies/mL. Similar findings were reported from Tanzania^38^ and South Africa^39^ with 89.9% and 88%, respectively. These consistent results across diverse settings underscore the effectiveness of targeted ART programmes for adolescents as propagated by UNAIDS.^40^ However, to further reduce transmission among this age group, adopting the more rigorous viral suppression threshold of < 50 copies/mL, referred to as ‘fully suppressed’ and recommended by the WHO, would be a critical consideration.

Of concern in Lusaka is that 15% of adolescents on ART in our study did not have VL results recorded, potentially underestimating or overestimating the true viral suppression levels within the population. This missing VL data may stem from delays in sample transport, missed data entries, or challenges with patient follow-up and adherence to monitoring schedules. Such issues have significant health system implications, including delayed interventions, undermined data-driven decision-making, weakened public health surveillance, misallocation of resources, and fragmented care.

Additionally, our study revealed that older adolescents aged 15–19 years were at greater risk of viral non-suppression compared to those aged 10–14 years, a finding consistent with other studies that link this disparity to challenges in transitioning from paediatric to adult care, which can disrupt continuity of care, reduce adult or caregiver support, and exacerbate adherence issues. Furthermore, adolescence is a period of significant physical, emotional, and social changes that can negatively impact treatment adherence, compounded by treatment fatigue among older adolescents who have been on therapy since early childhood, making it increasingly difficult to sustain adherence over time.^41,42^ Similar findings were reported in South African studies in the Cape Town Metro in the Western Cape Province and Ehlanzeni District in Mpumalanga.^14,43^

The majority of adolescents currently receiving ART in this study were on first-line treatment (DTG-based) and were more than five times more likely to have viral suppression than those receiving second-line treatment. The high viral suppression rate observed in the current study may be related to the successful introduction of a DTG-based regimen as part of the first-line regimen for all PLWH. This finding is supported by several other studies that reported that the DTG-based regimen is more effective in suppressing VL than the non-DTG-based regimen.^44,45,46^

Our study found that an optimal adherence rate (of > 95%) was significantly associated with viral suppression. Adherence may have been higher because pharmacy refills based on the EMR were used to assess adherence. However, research suggests that in resource-limited settings, pharmacy refill records correlate well with HIV outcomes and provide a more accurate measure of adherence than patient self-report and clinic-based pill counts.^47^

### Limitations

This study is a retrospective cohort analysis based on patient data routinely collected during clinic visits and interactions. As a result, the accuracy and completeness of the data available for extraction depended largely on the robustness of data collection and the thoroughness with which health workers and data entry clerks recorded individual client information. Certain variables, such as CD4 count, pregnancy status, and breastfeeding, had high levels of missing data, resulting in insufficient entries to reliably include them as covariates in the analyses. This incompleteness impacted the study’s ability to fully assess the influence of these variables on patient outcomes.

Despite possible limitations, such as missing or incomplete data, routinely collected and tracked data provide a real-time view of service delivery and patient outcomes, supporting timely decision-making and improvements in care. This approach is cost-effective, reduces the need for additional data collection and reflects the everyday reality of healthcare. It enables trend assessment, progress tracking, and identification of emerging issues. Finally, both operational strengths and weaknesses are highlighted while assessing the impact of interventions and system gaps.^48^

## Conclusion

Viral suppression among adolescents on ART was relatively high in this study in comparison to some regions, but it was still below the new target of 95% set by the UNAIDS. The reported low retention in care rates found in our study warrant further investigation and possible intervention. Older adolescents (15–19 years), those on second-line regimens, and non-adherent adolescents were found to be at higher risk of viral non-suppression. The results highlighted the need for targeted interventions focused on adolescent- friendly services which may include age-appropriate health services, psychosocial support which includes mental health services and disclosure and intentional transition processes from paediatric to adult care.
